# Trends in Paper-Based Sensing Devices for Clinical and Environmental Monitoring

**DOI:** 10.3390/bios13040420

**Published:** 2023-03-25

**Authors:** Shekher Kummari, Lakshmi R. Panicker, Jagadeeswara Rao Bommi, Sampath Karingula, Venisheety Sunil Kumar, Kuldeep Mahato, Kotagiri Yugender Goud

**Affiliations:** 1 Department of Chemistry, Indian Institute of Technology Palakkad, Palakkad 678557, Kerala, India; 2 School of Medicine, Case Western Reserve University, Cleveland, OH 44106, USA; 3 Department of Chemistry, National Institute of Technology, Warangal 506004, Telangana, India; 4 Department of Physical Sciences, Kakatiya Institute of Technology and Science, Warangal 506015, Telangana, India; 5 Department of Nanoengineering, University of California, La Jolla, San Diego, CA 92093, USA

**Keywords:** biosensors, clinical diagnostics, environmental monitoring, paper-based sensing devices, electrochemical sensors, optical sensors

## Abstract

Environmental toxic pollutants and pathogens that enter the ecosystem are major global issues. Detection of these toxic chemicals/pollutants and the diagnosis of a disease is a first step in efficiently controlling their contamination and spread, respectively. Various analytical techniques are available to detect and determine toxic chemicals/pathogens, including liquid chromatography, HPLC, mass spectroscopy, and enzyme-linked immunosorbent assays. However, these sensing strategies have some drawbacks such as tedious sample pretreatment and preparation, the requirement for skilled technicians, and dependence on large laboratory-based instruments. Alternatively, biosensors, especially paper-based sensors, could be used extensively and are a cost-effective alternative to conventional laboratory testing. They can improve accessibility to testing to identify chemicals and pollutants, especially in developing countries. Due to its low cost, abundance, easy disposal (by incineration, for example) and biocompatible nature, paper is considered a versatile material for the development of environmentally friendly electrochemical/optical (bio) sensor devices. This review presents an overview of sensing platforms constructed from paper, pointing out the main merits and demerits of paper-based sensing systems, their fabrication techniques, and the different optical/electrochemical detection techniques that they exploit.

## 1. Introduction

In today’s world, we are witnessing innumerable health-related issues and diseases silently co-existing with us. Providing equitable healthcare to all the worldwide population is the practice of Global Health. There are numerous factors that determine global health such as environmental safety, water and food quality, and disease control. Especially the unrestrained spread of hazardous contaminants due to industrial activities or microorganisms is a major challenge in many countries in the world. These contaminants can impede the functions of the ecosystem in any particular area by spreading in water, soil, and air and can enter the human life cycle through respiration, consumption, and skin absorption [1]. The influence of these contaminants on humans can vary depending on the degree of toxicity and contamination exposure period. Exposure can lead to headaches, nausea, lung failure, poisoning of the blood, kidney failure, and can even lead to cardiovascular failure. Hence, the development of diagnostic methods for environmental and clinical samples is an important priority for exploration and provides ample opportunities for researchers. 

Environmental and clinical sample screening is majorly dependent on the traditional, gold standard, sophisticated instruments such as gas/liquid chromatography, spectroscopic techniques, and enzyme-linked immunosorbent assay (ELISA). These methods give sensitive accurate and valid results. However, they are time-consuming, expensive, require high maintenance costs and lack portability. In addition, they consume large amounts of reagents, and experienced professionals are required to execute and analyze the data and results. To overcome these drawbacks, researchers are focusing on the development of affordable, accurate, rapid, robust, onsite, portable, and simple detection methods. Alternatively, biosensors, especially optical and electrochemical-based screening methods, are emerging, especially portable/wearable sensing strategies for the detection of various clinical [2,3], pharmaceutical [4,5,6,7] and environmental analytes [8,9,10]. These sensing methodologies are mainly developed on solids (glassy carbon, gold electrodes) [5], strips [11,12,13], textiles (screen printed electrodes) [14,15], paper [16], and as tattoo (epidermal) platforms [17] for various analytes. Among these, paper-based biosensors are rapidly growing in medical and environmental screening.

Paper-based biosensors are one of the alternative approaches for diagnosing diseases, pathogen detection, and monitoring patient health condition [16]. These sensing methods allow us to create simple, portable, and flexible diagnostic devices at an affordable price [18,19,20]. This fast and accurate sensing technology uses paper (which is hydrophilic) as a substrate that can easily develop and make hydrophobic microfluidic channels via patterning, thus allowing the analysis of sweat, tear, saliva, urine, and blood for various biomarkers/drugs, as well as water, air and industrial effluents for various toxic analytes. The most common beneficial properties of these paper-based biosensors are capillary action, adsorption, high surface to volume ratio, and better immobilization of biomolecules, such as antibodies, aptamers, and proteins, through their functional groups. Moreover, the paper-based approach enables easy disposal and allows the absorption and transport of reagents within the paper substrate, thereby avoiding the requirement for reagents to be handled by users [21,22]. Currently, on-site paper-based sensing strategies and portable devices are emerging for point of care detection [23]. For example, there are portable electrochemical sensing methodologies for on-site detection of pesticide residues in fruits and vegetables [24,25] and advances in optical-sensing strategies for the on-site detection of pesticides in agricultural foods [26].

In the last few years, there have been many reports in the literature on paper-based sensors and devices for clinical and environmental applications. Ataide et al. wrote a critical review on the development of electrochemical paper-based analytical devices over the last ten years that discussed the all aspects of paper-based devices, including procedures for fabricating conductive tracks on paper surfaces (screen printing, stencil printing, inkjet printing, pencil drawing, vacuum filtration, drop-casting, sputtering and wire placement etc.) and the development of different types of nanobiosensors [27]. Zhang et al. investigated the research progress and described the future trends of microfluidic paper-based analytical devices for in-vitro diagnosis [28]. In another review, Wang et. al. has critically examined the development of different microfluidic paper-based analytical devices for food contaminant detection [29]. 

**Figure 1:**
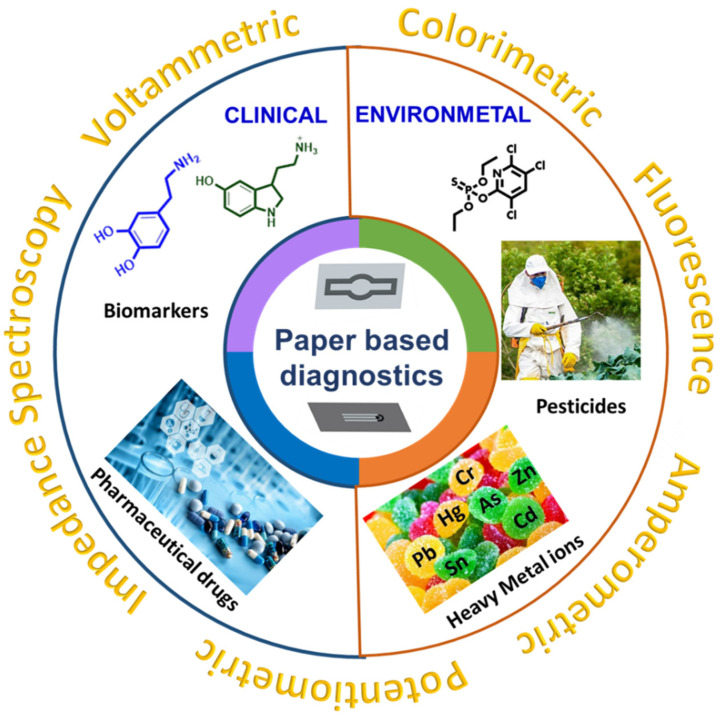
Clinical and environmental applications of paper-based sensors.

This present review provides an overview of sensing platforms constructed on paper, including the main merits and demerits of paper types, and the different fabrication techniques for integration of biorecognition elements on paper with nanomaterials. We mainly focus on electrochemical-based detection techniques (voltammetry, amperometry, electrochemical impedance spectroscopy, and potentiometry) and optical detection techniques (colorimetry, fluorescence, luminescence, and chemiluminescence paper-based biosensors) for the determination of various clinical and environmental samples. Figure 1 provides an overall summary of this review of clinical and environmental paper-based sensors and their transduction methods. 

## 2. Optical Paper-Based Sensors

The principal mechanism involved with optical biosensors is the interaction of diffraction light with the free/labelled target molecules. For example, a fluorescence-based device detects the change in frequency of electromagnetic radiation emission which is caused by the previous absorption of radiation of the target molecule. 

In the last two decades, there has been tremendous growth in optical-based biosensors for detection of various analyte molecules, such as clinical biomarkers, environmental pollutants, and agricultural toxins. This present section mainly discusses the recent developments in paper-based diagnostic devices based on optical transduction techniques such as fluorescence, colorimetric and absorbance. Our discussion mainly focuses on the fabrication protocol of the sensors, their principal mechanisms, analytical performance and significant possible real-world applications. 

### 2.1. Fluorescence 

Fluorescence biosensors have a wide range of applications in fields such as medical diagnostics, environmental monitoring, and food safety. They are particularly useful in the detection of small molecules and biomolecules, such as enzymes and DNA sequences. They are also used in drug discovery and development, where they can be used to screen large libraries of compounds for their ability to bind to a target molecule. A sensor strip based on paper containing carbon nanodots (C-dots) conjugated with a rhodamine moiety was formulated to demonstrate the usage of C-dots in the determination of metal ions. Yujun Kim et al., inspired by the unique features of C-dots, proposed a device using C-dots acting as an energy donor and the rhodamine moiety as an energy acceptor centre for the modulation of Förster resonance energy transfer (FRET) to detect Al^3+^. The rhodamine moiety was arrested on the C-dots surface by covalent coupling and is used for the specific target recognition. The authors characterized the prepared materials with UV emission, FTIR, XPS, TEM, and fluorescence emission spectroscopy. They observed that the C-dots-R6G system is more practical and efficient for Al^3+^ determination than a solution-based system as it uses commercial filter paper [30].

A paper-based aptasensor has been developed utilizing the advantages of the FRET procedure for rapid and sensitive determination of Pb^2+^ ions. The authors described a turn-on paper FRET aptasensor using a graphene oxide nanoquencher to detect Pb^2+^ and reported that the sensing platform exhibits linear ranges of 5–70 pM and 0.07–20 nM with an LOD of 0.5 pM within about 10 min. They fabricated the aptasensor on a sheet of chromatographic Whatman paper No. 1 which was modified with aptamer solution followed by GO solution [31].

Peroxide-based (H_2_O_2_, PBEs) explosives were sensitively detected by a curcumin-derived paper-based analytical device designed by Ling He et. al. The cellulose-based curcumin derivative sensor [ch][Cu] detects peroxides via the interaction between the former and phenol units, thereby giving an optical response. The excellent fluorophore nature of curcumin allowed the sensor to be developed to the level of a ‘just drop the sample’ type detection. Its unique properties such as low cost, ease of manufacturing, high stability made the sensor suitable for on-site detection of PBEs.

**Figure 2:**
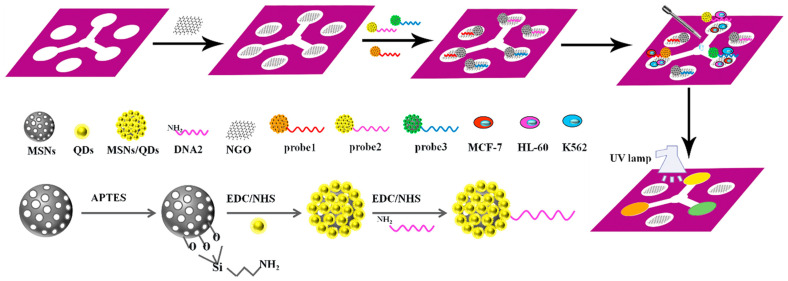
Fabrication procedure for a cyto-sensor on μ-PADs and the entire assembly process of the probe. Reproduced with the permission of [32]. Copyright 2016, Elsevier.

A microfluidic paper-based sensor containing MSN/QD-labelled aptamer for visual detection of multiplexed cancer cells has been developed. This sensor ensures low cost, simple, real-time detection of cancer cells (Figure 2). The fluorescence of these bioconjugates MSNs/QDs-DNA is quenched with graphene oxide to create a probe for cancer cell capturing. There is an increase in fluorescence as the probe is exposed to the cancer cells due to the release of colored probe; thus, the existence of target cells can be identified. The proposed sensing system exhibited a very high specificity due to the immobilized aptamers on the surface of NP [32].

Aniline-functionalized graphene quantum dots (GQDs) for glucose detection were utilised in a paper and fiber optic-based fluorescence sensor. These QDs were synthesized using microwave-assisted pyrolysis of fructose and characterized with TEM, XPS, UV-visible, FTIR, and Raman spectroscopies. Later the GQDs are modulated using phenyl boric acid (PBA), and this GQDs/PBA composite system works as a fluorescent turn-on sensor for glucose monitoring. The present a-GQDs/PBA fluorescent sensor showed a clear response toward glucose detection due to the specific interaction of PBA with glucose. The sensor was fabricated by printing the GQD ink, giving rise to a visual glucose detector. The sensor exhibited an LOD of 2.1μM with a high reproducibility [33].

A wearable smartphone-coupled microfluidic cotton thread device was developed for glucose detection in sweat samples. Sweat was acquired using a plasma treated hydrophilic cotton thread which acted as a microchannel. This smartphone-coupled device was further combined with an arm guard to show its response for non-invasive on-site detection of glucose in sweat samples. The device had a detection range of 50–250 μL with an LOD of 35 μL toward glucose in sweat [34].

**Figure 3:**
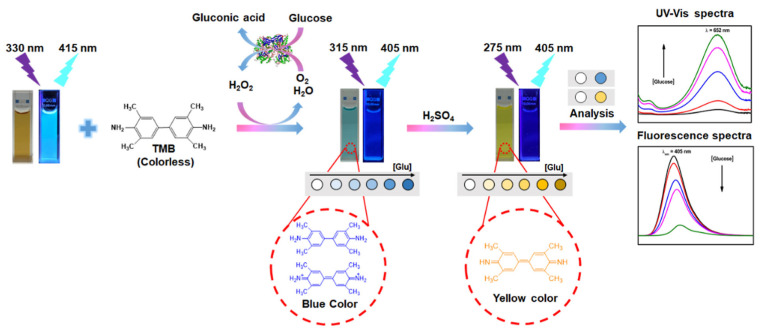
Schematic representation of metal oxide hybrid with N-doped carbon dots for the colorimetric detection of hydrogen peroxide and glucose. Reproduced with the permission of [35]. Copyright 2021, Elsevier.

Nitrogen-doped carbon dots and hybrid metal oxide structures were used to develop a paper-based fluorescence and colorimetric glucose sensor (Figure 3). In the presence of H_2_O_2_, the material exhibits intrinsic peroxidase-like activity and is used as a catalyst to oxidize 3,3′,5,5′-tetramethylbenzidine (TMB) to blue-emitting oxidized TMB (oxTMB) instead of GOx. The method of detection is based on the fluorescence of glucose and H_2_O_2_, and the sensor exhibits an LOD of 84 nM and 0.41 μM for H_2_O_2_ and glucose, respectively. Integration with a smartphone makes this sensor ideal for more for practical applications [35].

### 2.2. Absorbance and Colorimetric Sensors

Li et al. have demonstrated a paper-based sensor for sulfur dioxide detection in wine utilizing a colorimetric or surface enhanced Raman spectroscopy (SERS) dual-sensing strategy [36]. The authors observed that the SERS approach for SO_2_ exhibited good performance in a wide concentration range (1 M to 2000 M), with a limit of detection of 1 M. This dual-sensing strategy was revealed as an effective approach for developing a paper-based sensor for a variety of field-testing applications.

A paper-based colorimetric sensor for the monitoring of trace-level copper ions using thiosulfate catalytic etching of silver nanoplates was demonstrated by Chaiyo et al. [37]. The sensing system exhibited promising analytical performance for copper ion detection in a wide concentration range from 0.5 to 200 ng mL^−1^ with a LOD of 0.3 ng mL^−1^. Additionally, the sensor was further tested for its the trace level detection of copper in various samples such as blood, food and water.

Lookadoo et al. described the design and fabrication of paper-based optode devices (PODs) for K^+^ ions sensing in physiological fluids [38]. It was identified that this integrated prototype measurement system provides significant advantages compared with typical optode membranes and other paper-based methods in terms of being simple to use and cost-efficient, and potentially allowing for more effective disease management via telemedicine.

Qin et al. have fabricated a simple and eco-friendly paper-based sensor for chlorine detection [39]. The fabrication was carried out by simple hand-drawing of the poly(3,4-ethylenedioxythiophene): poly(styrenesul-fonate) (PEDOT: PSS) chemoreceptor as the sensing element on a paper strip. It was found that the developed sensor was more effective compared with existing colorimetric free chlorine strips. The sensor exhibited sensing across a wide range of concentration (0.5–500 ppm), which is ideal for monitoring for all types of water use.

**Figure 4:**
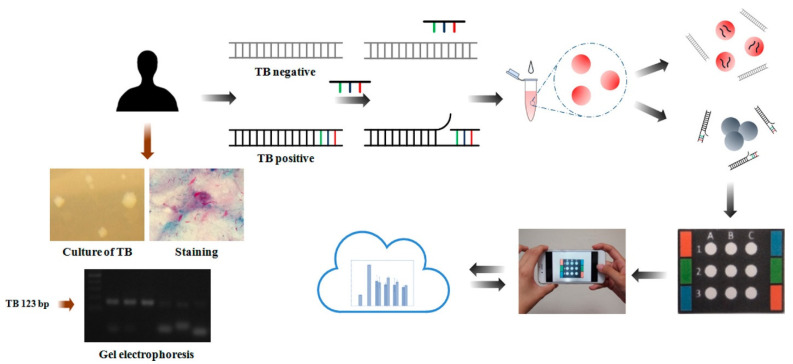
Schematic illustration of gold nanoparticle-assisted colorimetric sensor system using a paper-based analytical device for tuberculosis diagnosis. Reproduced with the permission of [40]. Copyright 2017, American Chemical Society.

Tsai et al. have designed a paper-based device for the monitoring of tuberculosis (TB) [40] (Figure 4). The authors were able to see the color change of AuNPs upon hybridizing single-stranded DNA probe molecules by targeted double-stranded TB DNA. They investigated the sensing system in various environments including the temperature denaturing and time at high temperature for various oligonucleotide probe sequences. Under optimized conditions, the sensing system exhibited a LOD of 1.95 × 10^−2^ ng mL^−1^ for TB DNA.

Bhattacharjee et al. have demonstrated the design and fabrication of mobile-based POC lung function monitoring which could be used to detect and treat chronic obstructive pulmonary diseases (COPDs) such as bronchitis and pneumonia. The nano-enabled humidity sensor was constructed by deposition of Au nanoparticles and CdS nanoparticles on a paper surface [41]. The mechanism involved in this sensor is as follows. During forced exhalation, the water molecules in the humid air are adsorbed, leading to them condensing on the paper-sensor. This lowers the electrical resistance of the sensor, which is then transformed into an electrical signal that is observed in the output. The current device results were verified to be as effective as several commercially available devices. 

Mustafa et al. designed a paper-based biosensor for determining the freshness of seafood, especially by the detection of hypoxanthine, a compound that indicates the rate of meat and fish degradation [42]. The developed biosensor quantifies the hypoxanthine, which is resealed upon degradation of fish/meat with xanthin oxidase enzyme. The fabricated portable biosensors are more cost-effective since they include all the reagents needed for analysis. The designed colorimetric sensor has tested for degradation in real fish and meat samples over a range of concentrations from 117 (±9) to 198 (±5) μM for 24 h.

**Figure 5:**
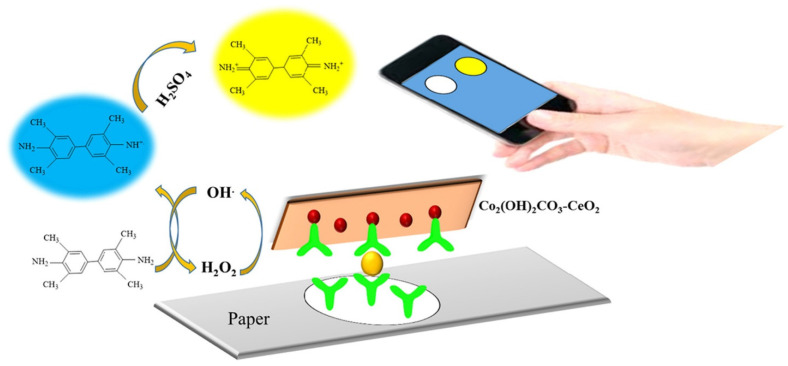
Graphic illustration and assay procedure for CEA detection using the microfluidic paper-based analytical device. Reproduced with the permission of [43]. Copyright 2018, Elsevier.

Alizadeh et al. have demonstrated a paper-based microfluidic device for the monitoring of tumor marker carcinoembryonic antigen (CEA) using the Co_2_(OH)_2_CO_3_-CeO_2_ catalyst (Figure 5) [43]. The immunosensor exhibited good performance in the linear CEA detection, ranging from 0.002 to 75.0 ng mL^−1^ with an LOD of 0.51 pg mL^−1^. This immunosensor was further tested using human serum for the screening of CEA and a high sensitivity similar to techniques such as ELISA was observed.

Kitchawengkul et al. have successfully developed a paper-based sensor for the detection of total cholesterol (TC) from whole blood with aid of nitrogen-doped carbon dots (N-CDs) coupled with a 3D microfluidic platform [44]. This sensor functions as a simple, cost-effective, sensitive and selective alternative for TC determination in blood samples, when compared with conventional colorimetric measurements.

A 3,3,5,5-tetramethylbenzidine (TMB) and CdSe/ZnS quantum dots (QDs) were assimilated into a filter paper to develop a paper-based colorimetric device for Hg^2+^ detection in tap water by Li et al. [45]. Here, the CdSe/ZnS QDs works as a photocatalyst to oxidize TMB, and the paper turns blue in the presence of visible light. Furthermore, Hg^2+^ can also increase the photocatalytic activity of CdSe/ZnS QDs, which increases the intensity of the paper color. The paper-based device color change can be observed immediately with the naked eye, or it can be photographed with a smartphone and subjected to quantitative analysis. In addition, a new microfluidic paper-based analytical device has been designed using an (Ag-µPAD)-based chemiresistor composed of silver ink for the sensitive and quantitative determination of nitrite ions in environmental analysis [46]. This paper-based sensor was effectively employed in a real-world application of the sensor system for the detection of nitrite in tap, river and lake water samples. Yahyai et al. developed an S,N-doped carbon quantum dot paper-based chemiluminescence detection method for the specific determination of bendiocarb [47]. This is a carbamate pesticide and its detrimental effects on both people and the environment have made the need for low-cost, portable, and simple-to-use analytical devices imperative. Recent studies explored and analyzed a paper-based analytical device (PAD) with a chemiluminescence (CL) sensing platform for the quick, dependable, and sensitive bendiocarb detection. Grazioli et al. demonstrated a deep-eutectic solvent (DES)-soaked colorimetric paper-based sensor for malondialdehyde (MDA) detection [48]. MDA is a byproduct of polyunsaturated fatty acid peroxidation that is commonly used as a target for determining the oxidation state of food. Their study established that this reaction also takes place in a DES, such as choline chloride-malonic acid (ChCl-MA), which exhibits inherent acid characteristics, and that it does so in a manner that is analogous to other DESs. This enabled the development of an MDA detection method suitable for colorimetric smart labels that might be used on packaged foods. A 3D network polymer hydrogel paper-based microfluidic device was developed by He et al. [49] for glucose screening in whole blood. A sample input zone and a detection zone are located on an analytical device that is shaped like a mushroom in the suggested approach. Plasma diffuses into the detection zone when blood is dropped onto a µPAD inlet section, and a metallic 3D polymer hydrogel vehicle is implanted in the detecting region. A copper complex reacts to oxygen changes, and glucose oxidase (GOx), which is trapped inside the gel as a bioactivity preservative, acts as the gel vehicle. Using control and actual whole blood samples with glucose from 3 to 200 mM, the validity was established and the detection results were shown to be consistent with those found using a glucometer, indicating the possible real-time application of the sensor device.

The properties of optical sensors of various paper-based sensing devices are compiled in Table 1. This table includes detailed information on the analytical method, sensing platform, material interface, sensor sensitivity, and real demonstrations. This literature indicates that the nanomaterial interfaced paper-based biosensors exhibit better performance, due to the better immobilization which enhances the physical and electrical properties of the sensing assay. 

## 3. Electrochemical Paper-Based Sensors 

The basic principle of electrochemical biosensors is that chemical reactions between the immobilized bio or synthetic recognition element and the target analyte produce or consume ions or electrons, which affects the measurable electrical properties (such an electric current or potential) of the electrode–electrolyte interface. These electro-analytical properties can be measured with the several types of electrochemical techniques such as voltammetry, amperometry and electrochemical impedance spectroscopy. 

Electrochemical paper-based sensors are devices consisting of a paper substrate, typically made of cellulose, which is coated with an electrode material such as gold or carbon. The electrode material is functionalized with a specific receptor, such as an antibody or enzyme, that can selectively bind to the target analyte. When a sample containing the analyte contacts with the sensor probe, the receptor on the electrode selectively binds to the specific target analyte and generates an electrical signal that can be measured by the analyzer. The magnitude of the electrical signal is proportional to the concentration of the target analyte in the sample. Electrochemical paper-based sensors have several advantages over traditional sensors, including low cost, ease of use, portability, and the ability to detect multiple analytes simultaneously. They also have potential applications in various fields, such as healthcare, environmental monitoring and food safety. 

**Figure 6:**
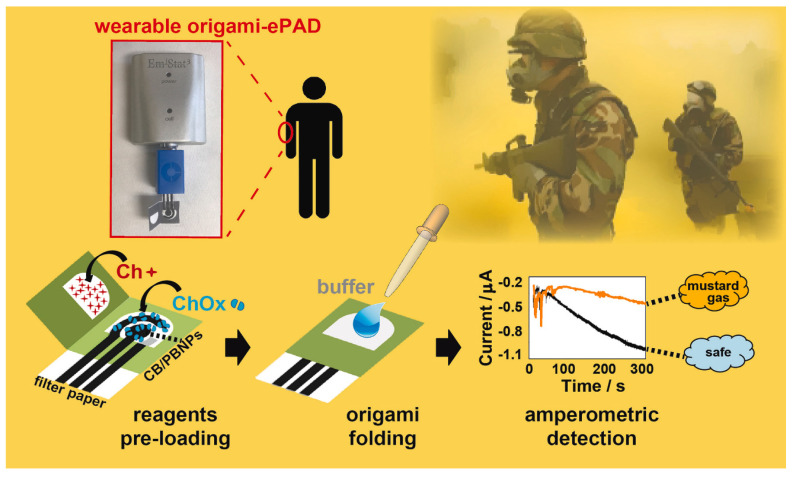
Schematic representation of working principle of the wearable origami-paper-based sensor and its on-site detection of mustard agents for security field application. Reproduced with the permission of [107]. Copyright 2019, Elsevier.

Noemi et al. fabricated a novel wearable paper-based biosensor for the on-site detection of mustard agents for security field applications (Figure 6). In their study, the authors monitored mustard gas and its inhibitory effects on the choline oxidase enzyme by recording the amperometry for the enzyme byproduct H_2_O_2_. A filter paper support is used for the screen-printing of the electrodes. This allows binding between the porosity of the paper and the pre-loaded required components into a network of cellulose to develop an origami-link reagent-free device. A carbon black/PB modified graphite ink was used for the WE for H_2_O_2_ reduction. The proposed sensor is capable of measuring the mustard agent in the aerosol phase and liquid phase directly. Moreover, the use of the biosensor was confirmed for real-time determination of sulfur mustard with a detection limit of 1 mM and 0.019 g min/m^3^ for the liquid and aerosol phases [107].

Meng Li and his research group proposed a low-cost highly integrated sensing paper (HIS) using 2D Ti_3_C_2_T_x_ MXene as the active material and foldable paper as a sweat sensing patch for monitoring glucose and lactate. The sensor was assembled with the HIS paper, hydrophobic protecting wax, conducting electrodes and the incorporated Mxene/methylene blue (Ti_3_C_2_T_x_/MB) active materials by using a simple printing process followed by folding into a three-dimensional (3D) structure. Here, the Ti_3_C_2_T_x_ MXene single flake-like structure improves the biorecognition element immobilization process. In addition, the inclusion of MB with Ti_3_C_2_T_x_ MXene helps enhance electron transfer and charge migration, which leads to better electrochemical sensing for real-time screening of glucose and lactate. The sensor exhibited high sensitivity towards them, with a sensitivity of 2.4 nA µM^−1^ and 0.49 µA mM^−1^ for glucose and lactate, respectively [108]. 

Caio et al. synthesized magic-size CdSe/CdS quantum dots to develop a paper-based electrochemical device (PED) for dopamine sensing. A simple and low-cost three-electrode novel PED was developed on a drawn tracing paper substrate. The graphite working electrode was modified with the magic-size CdSe/CdS quantum dots (MSQDs) to enhance the electrocatalytic response. The surface morphology of the designed electrode was well characterized in SEM and EIS studies. The DPV technique was assessed in sensitivity studies which found a low detection limit of 0.096 µmol L^−1^ within the linear range of 0.5 to 15 µmol L^−1^ for the detection of dopamine. The selectivity of the sensor device was tested in the presence of KCl, NaCl, acetaminophen (AC), ascorbic acid (AA), and uric acid (UA). Furthermore, the CdSe/CdS quantum dots modified sensor device was tested with blood serum samples for the detection of dopamine [109]. 

A disposable paper-based electrochemical analytical devise was designed with a multiplexed working electrode system for the detection of creatinine, glucose and uric acid. The device has a sample injection hole connected with four components constructed with 16 microfluidic channels and 16 WEs. In this multiplexed determination system, each WE is uniquely modified with respect to its target analyte detection. For example, a glucose oxidase-modified electrode with ferrocene carboxylic acid mediated electron transfer for glucose detection, and a carbon black nanoparticle-modified working electrode was used to improve the anodic peak current response for uric acid detection. The paper-based electrochemical device provided limits of detection of 0.120 mmol L^−1^ for glucose, 0.084 mmol L^−1^ for creatinine, and 0.012 mmol L^−1^ for uric acid. Furthermore, the multiplexed sensor exhibited good performance in detection of the three analytes in real urine samples [110]. 

An inexpensive paper-based electrochemical device was fabricated based on the principle of target-induced confirmation switching of an electrochemical label-aptamer link for the detection of DNA and thrombin. The device originally based on the slip chip concept was reported to be reliable, and later, it was adapted to the paper platform. The base layer sensor was constructed by printing a wax pattern on both sides of the chromatography paper and punching a hole in the base layer to expose the working electrode fully on the device. Then three carbon electrodes were printed on the wax-patterned paper. On the working electrode, the carbon surface was electroplated by Au to simplify the surface immobilization of the DNA probe, and the sensor exhibited detection limit values of 30 and 16 nM for DNA and thrombin, respectively. Moreover, the sensor exhibited a long shelf life of 4 weeks without losing its sensitivity [111]. 

Moazeni et al. developed an impedimetric electrochemical sensor on a peptide-modified point-of-care paper device for the detection of alpha-fetoprotein in human serum. In this device, peptide-modified microfluidic plastic-paper chips were constructed in three different layers consisting of a lower layer of silver–graphene decorated cellulose paper on a flexible plastic sheet and an upper layer of Diphenylalanine (FF) polymer. This FF layer helps to enhance the electrocatalytic performance of the sensor and involves antibody immobilization stability with an amine-aldehyde reaction. The specific monoclonal antibodies interact with the target alpha-fetoproteins (AFP) on the microchip surface. These interactions are monitored by electrical responses in the absence and presence of AFP. This electrical response varies with several parameters, including pH, the concentration of the FF solution, and cellulose fibers morphology, and these parameters were systematically optimized for protein detection. Under these optimum conditions for impedance measurements, AFP exhibited low detection limits of 1 and 10 ng mL^−1^ in PBS and plasma [112].

A microfluidic paper-based analytical device (µPAD) was fabricated for the determination of the biomarker p24 antigen for human immunodeficiency virus (HIV) using the electrochemical impedance spectroscopic (EIS) technique. A wax-printed chromatographic paper was heated to create a cramped hydrophilic area for the electrochemical reaction zone, and the reference and counter electrodes were formed in each reaction zone by silver/silver chloride and carbon inks screen printing. A zinc oxide nanowires (ZnO NWs)-modified working electrode was used to enhance the response of the EIS sensor towards the p24 antigen detection for HIV, and a detection limit of 0.4 pg mol^−1^ was observed. Moreover, these biosensors are capable of detecting SARS-CoV-2 in human serum [113].

Charles S. et al. demonstrated a multiplexed electrochemical paper-based analytical device (ePAD) for the simultaneous detection of three major cardiovascular diseases (CVDs) biomarkers: troponin (cTnI), procalcitonin (PCT) and C-reactive protein (CRP). The ePAD consists of wax-patterned paper stacking, laser-cut transparency double-sided adhesive film protecting the sample inlet, and fluidic channels connected to a separate detection zone for each analyte. A graphene oxide (GO)-modified electrode was used for immobilization of the antibodies for the target CVDs biomarkers on the ePAD, and the concentration of each biomarker was estimated using the square wave voltammetry (SWV) technique. In the presence of biomarkers, a significant current decrease was an observed in a concentration-dependent manner, but there was no current change in the absence of biomarkers. The sensor exhibits linearity over a wide range (R^2^ > 0.99) with LOD values of 0.38 ng mL^−1^, 0.16 pg mL^−1^, and 0.27 pg mL^−1^ observed for CRP, cTnI, and PCT, respectively [114]. 

**Figure 7:**
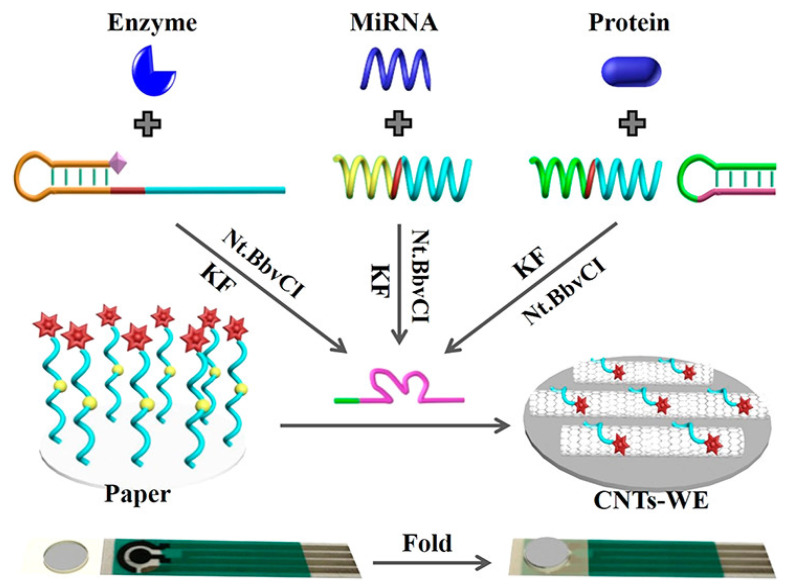
Schematic representation of a universal paper-based electrochemical sensor system that uses a paper modified with signal molecule-labeled DNA and a screen-printed electrode. Reproduced with the permission of [115]. Copyright 2019, American Chemical Society.

Liu et al. demonstrated a universal signal molecule-labeled DNA-modified paper-based sensor platform for the determination of various biomarkers such as miRNA, ALP, and CEA (Figure 7). The analyses depend on the target-induced Mg^+2^ synthesis-dependent DNAzyme for the substrate DNA cleavage from the paper, which was established by a microRNA recognized probe for miR-21, a DNA aptamer probe for carcinoembryonic antigen, and a phosphorylated hairpin probe for alkaline phosphatase. The unique advantage of the sensing system is selective zero-background detection, which is achieved through the specific target-triggered deposition and amplification of the DNAzyme-catalyzed signal. The use of this sensing system was also demonstrated in spiked serum sample screening, which could lead to the point-of-care detection of clinical samples in the future [115]. Wang et al. synthesized AuNPs-modified metal–organic frameworks (MOFs) by a strand displacement process to develop a highly sensitive paper-based biosensor for microRNA detection [116]. Cu-MOFs were used to develop a novel catalytic material that offers a larger surface area for the encapsulation of AuNPs and the immobilization of DNA strand 1 (S1). The generated S1-AuNPs@Cu-MOFs was connected to the modified electrode via chain hybridization in this sensing procedure, which showed promise for signal amplification. The released target was available for the beginning of several cycles. In the presence of glucose, AuNPs and Cu-MOFs are driven to catalyze the oxidation of glucose, resulting in a wide linear detection range from 1.0 fM to 10 nM, and an incredibly sensitive detection limit of 0.35 fM for miRNA-155.

Cinti et al. have developed Prussian blue nanoparticle (PBNPs) synthesized on a paper-based electrochemical biosensor for the detection of glucose in whole blood. In this article, the authors proposed a filter paper as a scaffold, and PBNPs were synthesized on the filter paper using just a few ml of their precursors without the use of any reducing agents or any external inputs in a demonstration of this unique approach. Using glucose oxidase as the biological recognition component, the eco-designed “Paper Blue” was paired with wax- and screen-printing to develop a reagentless electrochemical point-of-care device for diabetes self-monitoring [117]. In one more article, Li et al. demonstrated a one-pot co-reduction-based reduced graphene oxide paper with palladium modification that can function as a glucose sensor [118]. The synthesized Pd/rGO paper and used SEM, XRD, and Raman spectra to describe the structural composition of Pd/rGOP. By using chronoamperometry, the authors investigated the Pd/rGOP capability for glucose sensing, and the sensitivity was found to be as high as 6.70 µA mM^−1^ cm^−2^ at the linear range of 0.5 to 8 mM, indicating its potential as a reliable tool in point-of-care medical devices. Along with this, Narang et al. designed a point-of-care paper-based device with integrated micro fluidic zeolite nanoflakes and graphene oxide for electrochemical detection of ketamine [119]. The EµPAD has several benefits, including an easy method of use, affordability and commercialization possibilities. EµPAD augmented with nanocrystals demonstrated a broad linear range of 0.001–5 nM/mL, with an LOD of 0.001 nM/mL. The developed sensor showed a strong correlation (99%) when tested on real-time samples such as alcoholic and non-alcoholic drinks. The current fabricated device could undergo further development for industrial translation.

Electrochemical sensors of various paper-based sensing devices are compiled in Table 2 with detailed information about the analytical method, sensing platform, material interface, sensor sensitivity, and real sample demonstration. The literature indicates that the nanomaterial interfaced paper-based biosensors exhibit better performance, due to better immobilization and the enhanced conducting and catalytical properties of the sensing platform. 

## 4. Summary, Conclusions, and Future Perspectives

In this review, we have summarized various paper-based sensing platforms for detecting analytes of clinical and environmental safety importance. We have discussed the fundamental characteristics of various such sensors based on electrochemical and optical transducers, providing an overview in tables. This is a comprehensive summary of the analytical performances of the recently reported developments along with a brief discussion on the sensing principle, immobilization procedures, material interfaces, sensor performance, and real-time application demonstrations. The incorporation of nanomaterials in the transducers has enhanced the analytical performances of the paper-based sensors. While the developments in paper-based sensors are mainly targeted to developing low-cost modules, these are limited to qualitative determinations. Various strategies have been adopted to modify such qualitative sensing by incorporating image processing and controlled imaging modules. However, the modules reported are limited to the semi-quantification of the analytes. Future work should be directed toward the exploration of the quantitative estimation of analytes. Although paper-based sensors have seen widespread acceptance due to their advantages of being a low-cost material, they face various challenges in their integration into the analyzer modules. In addition, most of the sensors in commercial spaces utilize electrochemically based sensing, and unfortunately, paper-based sensing modules are limited. Future research will involve various studies to develop sensing modules that use paper substrates. In addition, studies on various challenges associated with the paper-based substrates in integrating electronic circuitry will open a new avenue in low-cost biodegradable quantitative biosensing paper-based modules. Further studies on polymer coatings will help improve the usability of paper as substrates in electrochemical formats. Such efforts will enhance the development of biosensors to enable the efficient detection of the proteinaceous targets of various diseases including diabetes, liver, and kidney disorders, etc. Furthermore, the mass fabrication of the device modules is a demanding task which may limit progress in the sensor development process. The efforts on improving the techniques for mass manufacturing and handling of the sensors would enable simple fabrication of the sensors on a commercial scale. 

## Figures and Tables

**Table 1:** Paper-based optical sensors for clinical and environmental applications.

No.	Nanocomposite|Electrode	Analyte	Method	Linear Range	LOD	Interferents	Real Sample	Ref.
**Clinical Samples**								
1	Cellulose-paper	BSA	SERS	10–60 mg mL^−1^	10 mg mL^−1^	-	-	[50]
2	PODs	K^+^	Colorimetric	2–7 mM	0.089 mM	Ca2^+^, Li^+^, Mg^2+^	plasma	[38]
3	Caffeine benzoate paper	Bilirubin	Colorimetric	0 to 25 mg dL^−1^	1.2 mg dL^−1^	spiked hemoglobin	jaundiced whole human blood samples	[51]
4	Wax screen printing	198-bp DNA	Chemiluminescent	1.94 × 10^−1^ pmol/L–1.94 × 10^4^ pmol/L	6.3 × 10^−2^ pmol/L	S1 + S2 + S3, S4 + S2 + S3, S1 + S2 + S5, S4 + S2 + S5 and S1 + *E*. *coli* + S3	-	[52]
5	Co_2_(OH)_2_CO_3_-CeO_2_	CEA	Colorimetric	0.002–75.0 ng mL^−1^	0.51 pg mL^−1^	AFP, CA 125, CA 15-3	human serum samples	[43]
6	Iron(III)-thiocyanate	Thiocyanate	Colorimetric	0.25 and 20 mM	0.06 mM	-	human saliva	[53]
7	PGA-RR	Polygalacturonase	Colorimetric	0.02–0.1 unit	0.02 unit	-	-	[54]
8	Tetrakis (4-octyloxyphenyl)porphyrin cobalt(II)	Thiocyanate	Colorimetric	0.001–5 mM	1.26 μM	SCN^−^, NO_2_^−^, AsO_3_ ^3−^, AsO_4_ ^3−^, ClO_4_^−^, NO_3_^−^, Br^−^, Cl^−^, H_2_PO_4_^−^, I^−^, F^−^, SO_4_^2−^.	urine samples	[55]
9	N-CDs	Cholesterol	Colorimetric	2.5–7.5 mM	0.676 mM	-	whole blood samples	[44]
10	AuNPs	Double-stranded TB DNA	Colorimetric	1.95 × 10^−2^–1.95 × 10^1^ ng/mL	1.95 × 10^−2^ ng/mL	-	-	[40]
11	Chitosan oligosaccharide lactate	Glucose Uric acid	Colorimetric	0–500 mg/dL 0–200 mg/dL	0.6 mg/dL 0.03 mg/dL	-	human urine	[56]
12	Paper-based ELISA	Ketamine	Colorimetric	10^−4^–10^−1^ mg/mL	0.03 ng/mL	-	oral fluid sample	[57]
13	Citrate-capped PtNPs	Uric acid	Colorimetric	0–8 mM	4.2 ± 5 µM	K^+^, Na^+^, Mg^2+^, Ca^2+^, Zn^2+^, Glu, DA, UA	human urine	[58]
14	5CB/LCs	Bilirubin	Distance-based	2.0–30.0 pmol/L	0.80 pmol/L	glucose, fruc- tose, lactose, sucrose, galactose, hemoglobin, bovine serum albumin, biotin, trypsin, cholesterol, glutathione, cysteine, glutamic acid, salicylic acid, ascorbic acid.	human control urine human serum	[59]
15	Zr-MOF/Fe_3_O_4_(TMC)/ AuNCs	HbA1c	ECL	2–18 0.072%	0.072%	-	human blood sample	[60]
16	Porous AuPd alloy	MCF-7	ECL	450–1.0 × 10^7^ cells mL^−1^	250 cells mL^−1^	HepG2, SK-BR-3	-	[61]
17	GO-aptamer	MCF-7 HL-60 K562	Fluorescence	180–8 × 10^7^ cells mL^−1^ 210–7 × 10^7^ cells mL^−1^ 200–7 × 10^7^ cells mL^−1^	6270 cells mL^−1^ 65 cells mL^−1^	-	-	[32]
18	MFNCDs	H_2_O_2_ Glucose	Fluorescent Colorimetric	0–15 mM 500 nM–15 mM	97 nM 0.85 μM 84 nM 0.41 μM	ascorbic acid, uric acid, L-cysteine, dopamine, glycine, sorbitol, glutathione	real serum	[35]
19	ISOs	Ca^2+^	Fluorescent	10^−5^–1 mol L^−1^	19.3 μmol L^−1^	Mg^+2^, Na^+^, K^+^	mineral water	[62]
20	a-GQDs	Glucose	Fluorescent	0.05–20 mM	2.1 μM	maltose, lactose, fructose, sucrose	live cells	[33]
21	P-BPE	H_2_O_2_ Glucose	Colorimetric	0.1 mmol L^−1^–4.0 mol L^−1^ 0.1–50 mmol L^−1^	4.9 μmol L^−1^ 70 μmol L^−1^	AA, UA	human serum samples	[63]
22	Cu complex/polyacrylamide	Glucose	Luminescent	3–200 mM	0.44 mM	-	whole blood samples	[49]
23	μTPAD	Glucose	Colorimetric	50–250 μM	35 μM	-	human sweat	[34]
24	NiFe_2_O_4_/Paper-Based ME	HSA	Gauss meter	10–200 µg mL^−1^	0.43 µg mL^−1^	UA, CRE, HGB, BSA, CEA	-	[64]
25	CdS/RGO/ZnO	miRNA-21 miRNA-122b miRNA-7f	PEC	0.5 fM–100 pM 0.5 fM–100 pM 10 fM–100 pM	0.32 fM 0.37 fM 3.8 fM	-	human serum	[65]
26	Silver halide particles	Cysteine Glutathione Homocysteine	Photoreduction	10–100 μM 10–100 μM 20–100 μM	10.0 μM 10.0 μM 7.5 μM	glutamine, glutamic acid, cystine, asparagine, aspartic acid, glycine, histidine, lysine, valine, alanine, and arginine	human blood plasma	[66]
27	DNA-AuNPs	miR-29a	Colorimetric SERS	18–360 pg μL^−1^	47 pg μL^−1^	-	-	[67]
28	APTMS-GA	H_2_O_2_, Glucose anti-PSA	Colorimetric	2.5–500 mM 0.5–30 mM 0.1–10 ng/mL	-	IgG, IgM, CEA, TNF-α	human serum	[68]
29	PPX-chromatography paper	Glucose Protein ALP ALT Uric acid	Colorimetric	-	25 mg dL^−1^ 1.04 g L^−1^ 7.81 unit per L 1.6 nmol L^−1^ 0.13 mmol L^−1^	-	-	[69]
30	GR/Au-PWE	DNA	ECL	4.0 × 10^−17^–5.0 × 10^−11^ M	8.5 × 10^−18^ M	-	human serum sample	[70]
**Environmental monitoring**								
31	Pd NPs/meso-C	H_2_O_2_	Colorimetric	5–300 μM	-	-	milk matrices	[71]
32	CdSe/ZnS quantum dots	Hg^2+^	Colorimetric	0.1–100 μM	0.09 µM	Ca^2+^, K^+^, Mn^2+^, Co^2+^, Cr^3+^ and Ni^2+^	tap water	[45]
33	Au NPs/CdS NPs	Humidity	-	-	-	-	-	[41]
34	Ag-μPAD	Nitrite	Colorimetric	10–3200 μM	6.2 × 10^−5^ µM	H^+^, Na^+^, K^+^, Ca^2+^, NH^4+^, F^−^, Cl^−^, Br^−^, BrO^3−^, IO^3−^, NO^3^ and SO_4_^2−^	tap, river and lake water samples.	[46]
35	Piezoelectric inkjet printer	AChE	Colorimetric	-	0.01 ng mL^−1^, 0.04 ng mL^−1^	-	-	[72]
36	S,N-doped carbon quantum dots	Bendiocarb	Chemiluminescence	0.1–10 µgmL^−1^	0.02 µgmL^−1^	-	water and juice samples	[47]
37	PEDOT:PSS/graphene	Chlorine	Chemiresistive	0.1−500 ppm	0.18 ppm	-	on-site water	[73]
38	CURN	Mercury	Colorimetric	0.5–20 µg mL^−1^	0.17 µg mL^−1^	NH_4_^+^, C_2_O_4_^2−^, CO_3_^2−^, HCO_3_^−^, Br^−^, F^−^, Ni^2+^, Cd^2+^, Mn^2+^Bi^3+^, Cr^3+^, Cu^2+^ and Zn^2+^	water samples	[74]
39	Cys A@AuNPs	Hg(II) Cu(II) ions	Fluorescence	0.001–1 ppm 0.001–1 ppm	0.001 ppm 0.1 ppm	Mn(II), Zr(IV), Ba(II), Mg(II), Sr(II), As(III), B(III), Al(III), V(III), K (I), Na(I),	-	[75]
40	AgNPs/PVA	Lead	Colorimetric	20–1000 μgL^−1^	8 μgL^−1^	Mg(II), Ca(II), Ba(II), Hg(II), Ni(II), Mn(II), As(III), Al(III), Cl^−^ and CO_3_^–^	industrial waste water	[76]
41	chromatographic paper foils	MDA	Colorimetric	2.5–20 μM	-	-	-	[48]
42	rGO/MPy-GNRs	SO_2_	Colorimetric/ SERS	1–2000 μM	1.45 μM	-	wine	[36]
43	Curcumin-based PADs	Boron	Colorimetric	0.2–0.8 mg/L	-	Cl^−^, PO_4_^3−^, SO_4_^2−^, K^+^, Na^+^, HCO_3_^−^, NO_3_^−^ and Fe^3+^	-	[77]
44	NBT	Hypoxanthine (HX),	Colorimetric	-	3.7 μM	D-glucose, L-lactic acid, and dopamine	fish samples	[42]
45	AgNPs	Mercury	Colorimetric	1 to 4 ppb	0.86 ppb	-	drinking water	[78]
46	Quercetin-capped AgNPs	Carbaryl, paraoxon, parathion, malathion, diazinon, and chlorpyrifos	Colorimetric	-	29.0, 22.0, 32.0, 17.0, 45.0, and 36.0 ng mL^−1^	K^+^, Na^+^, Ca^2+^, Mg^2+^, SO_4_^2−^, NO_3_^−^, I^−^, Br^−^, Cl^−^, S^2−^	tap water, apple juice, and rice	[79]
47	Paper-grafted thioglycolic acid Cu NP	Cu(II)	Colorimetric	0.1–10 mM	33 μM	Zn(II), Sn(II), Ni(II) Li(I), Pb(II), Fe(III), Ag(I), Cr(I), Cr(II),	-	[80]
48	µPADs	Cu^2+^, Fe^3+^, Cr^2+^, Ni^2+^, Hg^2+^, and Pb^2+^	Colorimetric	-	15 µM, 3.58 µM 0.5 µM, 0.8 µM 0.1 µM and 0.3 µM	-	tap water	[81]
49	Zn-TPP	NH_3_ and CO_2_	Colorimetric	-	-	HCl, NaOH, NaHCO_3_	-	[82]
50	Wax printing	Nitrite	Colorimetric	10–500 μM	-	-	-	[83]
51	(rGO)-PdAu	Pb^2+^	Colorimetric	0.5–2000 nM	0.14 nM	K^+^, Mn^2+^, Zn^2+^, Cu^2+^, Ca^2+^, Ni^2+^, Cr^2+^ and Cd^2+^	tap water and river water	[84]
52	Wax printer	Chlorpyrifos	Colorimetric	8.60 ppm	0 to 100 ppm	-	-	[85]
53	MIP@MOF	thiacloprid	Colorimetric	0.1–1.2 μM, and 1.2–10 μM	0.04 μM	acetamiprid, imidacloprid and dinotefuran	Lettuce, water and soil	[86]
54	Cellulose paper	As^3+^, Nd^3+^ and Br^-^	Fluorescence	11.53 nM, 0.65 nM and 11.25 nM	As^3+^ (0–150 nM), Nd^3+^ and Br^−^ (0–120 nM)	-	industrial waste water	[87]
55	EuD_4_TEA-Au NPs	Cyanide	Fluorescence	-	10^−2^–10^−12^ M	F^−^, N_3_^−^, H_2_PO_4_^−^, CH_3_COO^−^, Cl^−^, Br^−^, SO_4_^2−^ and NO_3_^−^	drinking water	[88]
56	[Ch][Cur]-coated cellulose paper	TATP, DADP	Fluorescence	-	0.0–1000.0 ppm	-	-	[89]
57	Calix[4]arene	La^3+^,Cu^2+^, and Br^−^	Fluorescence	0.88 nM for La^3+^, 0.19 nM for Cu^2+^ 0.15 nM for Br^−^	-	Zn^2+^, Cd^2+^, Fe^2+^, Fe^3+^, La^3+^, As^3+^, Nd^3+^, Zr^4+^, Ca^2+^, Ce^3+^, Li^+^, Ag^+^, Ba^2+^, Co^2+^, Hg^2+^, Na^+^	soil samples for La^3+^, blood serum for Cu^2+^ industrial water for Br^−^	[90]
58	RFID	Chloride	Fluorescent	10^−7^–10^−5^ M	2.35 × 10^−7^ M	Na^+^, K^+^, Ca^2+^	sweat	[91]
59	FMA	H_2_S	Fluorescent	17–67 ppb	3 ppb	-	-	[92]
60	GQDs	o- and p-nitrophenols	Fluorescent	0.30–60.0 and 0.20–40.0 µg/mL	0.07 and 0.03 µg/mL	-	tap water, river water,	[93]
61	Calix[4]arene	Mn^2+^, Cr^3+^ and F^-^	Fluorescence	0–120 nM	11 nM for Mn^2+^, 4 nM for Cr^3+^, 19 nM for F^-^	-	blood serum, industrial waste water	[94]
62	PANI/Ge	NH_3_	Transient	-	20 ppm	Ethanol, acetone, DMF and 2-propnal	-	[95]
63	CuNCs	H_2_S	Photoluminescent	2–10 μM	1 μM	SO4^2−^, SO_3_^2−^, PO_4_^3−^, CO_3_^2−^, Ac^−^, NO_3_^−^, NO_2_^−^, ClO_4_^−^,	springwater samples	[96]
64	Tyrosinase immobilized	BPA	Colorimetric	0.05–3.87 μg/g	0.28 μg/g	-	-	[97]
65	PEDOT:PSS	Chlorine	Colorimetric	0.5–500 ppm	-	NaOCl, CaCl_2_, MgCl_2_, ZnCl_2_, K_2_SO_4_, CuSO_4_, (NH_4_)_2_SO_4_, NaHCO_3_, Na_2_CO_3_	tap water and swimming pool water	[39]
66	PAN	Cu(II)	Colorimetric	0.1–30.0 mg L^−1^	0.06 mg L^−1^	Fe(III), Al(III), Zn(II), Cd(II), Pb(II), Ca(II), Mg(II), and Ni(II)	hot water	[98]
67	AuNPs)	Hg^2+^	Colorimetric	25–750 nM	50 nM	Co^2+^, Mn^2+^, Pb^2+^, Ca^2+^, Cd^2+^, Cu^2+^, Ni^2+^, Zn^2+^, Cr^3+^	pond and river water	[99]
68	AgNPls	Cu(II)	Colorimetric	0.5–200 ng mL^−1^	1.0 ng mL^−1^	K^+^, Cr^3+^, Cd^2+^, Zn^2+^, As^3+^, Mn^2+^, Co^2+^, Pb^2+^, Al^3+^, Ni^2+^, Fe^3+^, Mg^2+^, and Hg^2+^	water food and blood	[37]
69	H-rGO	NO_2_	Conductometric	1–10 ppm	-	O_2_,O_3_,CO_2_, CO, SO_2_, NOx andNH_3_	-	[100]
70	paper@QDs@Cu-IIP	Cu^2+^	Fluorescent	0.032–3.2 mg L^−1^	0.012 mg L^−1^	Na^+^, K^+^, Ca^2+^, Mg^2+^, Zn^2+^, Ni^2+^, Co^2+^, Cd^2+^, Hg^2+^, Pb^2+^ and Fe^3+^	lake water and sea water	[101]
71	Rhodamine-appended C-dots	Al^3+^	Fluorescent	1 × 10^−4^–1× 10^−2^ M	3.89 × 10^−5^ M	Mg^2+^, Cu^2+^, Co^2+^, Ni^2+^, Mg^2+^, Zn^2+^, Fe^3+^, and Fe^2+^	-	[30]
72	Tb^3+^/BSA–AuNC	Hg^2+^	Fluorescent	0, 0.005–7 μM	1 nM	Ag^+^, Ca^2+^, Co^2+^, Cu^2+^, Cd^2+^, K^+^, Na^+^, Ni^2+^, Fe^2+^, Hg^2+^, Mn^2+^, Mg^2+^	-	[102]
73	PdA_2_	H_2_S	Luminescent	8–110 ppb	2 ppb	NO_2_ and SO_2_	-	[103]
74	SWNT-PABS	NH_3_	Resistance	10–250 ppm	-	-	-	[104]
75	Pencil-drawn	NO_2_	Chemiresistor	0.05–5 ppm	-	C_2_H_5_OH, CH_3_OH, NH_3_, CH_3_COCH_3_	-	[105]
76	ZnO/AuNPs	PCP	Photoelectrochemical	0.01–100 ng mL^−1^	4 pg mL^−1^	aldrin, heptachlor, chlopyrifos	-	[106]

BSA—bovine serum albumin; SERS—surface-enhanced Raman spectroscopy; POD—paper-based optode devices; Ti3C2Tx/MB—MXene/methylene blue; PBNPs—Prussian blue nanoparticles; CEA—carcinoembryonic antigen; RR—ruthenium red; PGA—poly-galacturonic acid; N-CDs—nitrogen-doped carbon dots; PtNPs—Pt nanoparticles; AgNP/BDD—silver nanoparticle-modified boron-doped diamond; Pd/rGOP—palladium/reduced graphene oxide paper; ZnO NWs—zinc oxide nanowires; CV—cyclicvoltammetry; Zeo–GO—zeolites nanoflakes and graphene-oxide nanocrystals; 5CB—immobilizing 4-cyano-40-pentylbiphenyl; LCs—liquid crystalline molecules; 3D Au NPs/GN—3D Au nanoparticles/graphene; DPV—differential pulse voltammetry; Au@PdPt NPs—trimetallic dendritic Au@PdPt nanoparticles; Cu-MOFs-Cu-based metal–organic frameworks; PAD—paper-based analytical device; AC—acetaminophen; MSQDs—magic-sized quantum dots; FCA—ferrocenecarboxylic acid; 8-OHdG-8-Hydroxy-2′-deoxyguanosine; AFP—Alpha-fetoprotein; FF—Diphenylalanine; EIS—electrochemical impedance spectroscopy; PEDOT: PSS—poly (3,4-ethylenedioxythiophene): poly(styrenesulfonate); CEA—carcinoembryonic antigens; PSA—prostatic specific antigen; ECL—electrochemiluminescence; HbA1c—Glycated hemoglobin; MFNCDs—metal oxide hybrid with nitrogen-doped carbon dots; ISOs—carrier-based ion-selective optodes; a-GQDs—Aniline functionalized graphene quantum dots; μTPAD microfluidic thread/paper-based analytical device; HSA—human serum albumin; ME—magnetoelastic; PEC—photoelectrochemical; ISM—ion-selective membrane; DPS—disposable paper substrate; SWV—square wave voltammetry; CRP—C-reactive protein; cTnI—troponin I; PCT—procalcitonin; SERS—surface enhanced Raman scattering; APTMS-3—aminopropyltriethoxysilane; GA—glutaraldehyde; PPX—poly(chloro-p-xylene) 3. electrochemical paper-based sensors.

**Table 2:** Paper-based electrochemical sensors for clinical and environmental applications.

No.	Nanocomposite|Electrode	Analyte	Method	Linear Range	LOD	Interferents	Real Sample	Ref.
**Clinical Samples**								
1	MB/Ti_3_C_2_Tx/SPCE	Glucose and lactate	Amperometric	0.08–1.25 mM 0.3–20.3 mM	17.05 μM 3.73 μM	uric acid ascorbic acid	sweat	[108]
2	CuO/IL/ERGO	Creatinine	Amperometric	0.01–2.0 mM	0.22 μM	glucose, uric acid, urea, ascorbic acid	human blood serum	[120]
3	PBNPs	Glucose	Amperometric	up to 5 mM	0.17 mM	-	blood glucose	[117]
4	Aptamer-antibody	Gluten	Amperometric	0.2 and 20 mg L^−1^	0.2 mg L^−1^	-	corn flakes, chickpea flour signal	[121]
5	AgNP/BDD	Cholesterol	Chronoamperometric	0.01–7 mM	6.5 µM	glucose (Glu), ascorbic acid (AA), and uric acid (UA)	bovine serum	[122]
6	FCA/GOx/PAD	Glucose	Chronoamperometric	1–12 mM	0.05 mM	AA, DA, UA	blood samples	[123]
7	Pd/rGOP	Glucose	Chronoamperometry	0.5–8 mM	30 µM	UA, AA	-	[118]
8	ZnO NWs	Glucose	CV	0–15 mM	59.5 μM	-	human serum	[124]
9	anti-HCT-interference	Glucose	CV	3.7–13.8 mM	0.88 mM	-	whole blood samples	[125]
10	Carbon ink	Glucose	CV	4.4–6.6 mM	-	-	orange juice cola beverage	[126]
11	Zeo–GO	Ketamine	CV	0.001–5 nM/mL	0.001 nM/mL	whiskey urine organic juice	-	[119]
12	3D Au NPs/GN	K-562 cells	DPV	1.0 × 10^3^–5.0 × 10^6^ cells/mL	200 cells/mL	-	-	[127]
13	Au@PdPt NPs	K-562 cell	DPV	1.0 × 10^2^–2.0 × 10^7^ cells mL^−1^	31 cells mL^−1^	MCF7, H9c2, normal cells	human serum samples	[20]
14	Cu-MOFs/uNPs	miRNA	DPV	1.0 fM–10 nM	0.35 fM	nDNA, nRNA, tRNA, sRNA	serum samples	[116]
15	PAD	Uric Acid	DPV	0.1–1 mM	8 μM	urea, AA, glucose	human urine	[128]
16	CNTs-WE	miR-21	DPV	1 fM–1 μM	-	miR-141, miR-155, miR-199a, miR-143	spiked serum samples	[115]
17	CdSe/CdS MSQDs	Dopamine	DPV	0.5–15 μmol L^−1^	96 nmol L^−1^	AA, UA, AC	human blood serum	[109]
18	FCA carbon black	Glucose Uric acid	DPV Chronoamperometry	0.63–20.0 mmol L^−1^ 0.05–3.00 mmol L^−1^	0.12 ± 0.03 mmol L^−1^ 0.012 ± 0.002 mmol L^−1^	-	urine sample	[110]
19	Carbon ink/PEDOT	8-OHdG	DPV	50–1000 ng/ml	14.4 ng/ml	UA, AA	diluted serum samples	[129]
20	Graphene-Ag	AFP	EIS	1–10^4^ ng/ml	-	ascorbic acid, glucose	human serum	[112]
21	ZnO NWs	p24 antigen IgG antibody	EIS	-	0.4 pg mL^−1^ 10 ng mL^−1^	-	human serum samples	[113]
22	Graphene-PEDOT:PSS	CEA	EIS	0.77–14 ng mL^−1^	0.45 ng mL^−1^	BSA, PSA and insulin	serum sample	[130]
23	rGO-Au-SPEs	Glucose	EIS	3.3–27.7 mM	-	-	whole blood	[131]
24	CdS/RGO/ZnO	miRNA-21 miRNA-122b miRNA-7f	PEC	0.5 fM-100 pM 0.5 fM–100 pM 10 fM–100 pM	0.32 fM 0.37 fM 3.8 fM	-	human serum	[65]
25	ptCuMOFs/DNA	miR-141 miR-21	SWV	1 fM–1 nM	0.1 fM	uric acid, dopamine, miR-210	serum samples	[132]
26	BSA/rGO/antibody/GO/G-SPCE	CRP cTnI PCT	SWV	1–100,000 ng mL^−1^ 0.001–250 ng mL^−1^ 0.0005–250 ng mL^−1^	0.38 ng mL^−1^ 0.16 pg mL^−1^ 0.27 pg mL^−1^	glycine, creatinine, L-cysteine, homocysteine, albumin, hemoglobin, myoglobin	serum sample	[114]
27	Au-carbon	DNA thrombin	SWV	-	30 nM 16 nM	-	-	[111]
**Environmental monitoring**								
28	CFP	Nitrite	Amperometric	0.1–3838.5 μM	0.07 μM	NaNO_2_, KNO_3_, NH_4_Ac, MgSO_4_, Na_2_SiO_3_ and ZnSO_4_,	mineral water	[133]
29	CF/GO/cellulose	amitrole	Amperometric	0 mM to 0.4 mM	2.44 × 10^−7^ mol L^−1^	Cl, NaNO_3_, NaAc, K_2_SO_4_, urea, and glucose -	Tap and lake water	[134]
30	ERGO-AuNP-CC-Ag-PPPE	Ni^2+^	ACSV	50–500 µg L^−1^	32.19 µg L^−1^	-	drinking water	[135]
31	Paper CB-SPE	Phosphate	Chronoamperometry	4 μM	10–300 μM	-	river water	[136]
32	GNPs/graphene/MCE	Nitrite	DPV	0.1 µM	0.3–720 µM	Na^+^, Ca^2+^, Mg^2+^, K^+^, Zn^2+^, Cl^−^, NO_3_^−^, CO_3_^2−^	Lake water, river water and milk	[137]
33	(CuNP/SPGE)	NOx gas	DPV	0.23 vppm and 0.76 vppm	-	SO_2,_ CO, N_2_O and O_3_	-	[138]
34	Carbon Ag ink	3-nitrotyrosine	SWV	49.2 nM	500 nM–1 mM	tyrosine, ascorbic acid, uric acid and creatinine	saliva, blood and urine	[139]
35	MWCNTs/l-PAD	Fluorene	LSV	0–100 µM	0.0500 µM	-	-	[140]
36	Aptamer-Modified mPEDs	Cocaine	SWV	1−100 μM	1 μM	-	urine, saliva	[141]
37	ZnO NPs	Picric acid	SWV	4 μM–60 μM	4.04 μM	zinc, lead, copper and mercury	lake water	[142]
38	IP-SPE	NADH and nitrite	CV	10–100 μM 100–1000 μM	1.8 μM and 15.1 μM	-	-	[143]
39	Pencil graphite	p-nitrophenol	DPV	10–200 μM	1.1 μM	4-aminophenol, 1,4 dihydroxy benzene and phenol	-	[144]
40	Prussian blue nanoparticles	Atrazine 2,4-D paraoxon	Chronoamperometric	10–100 ppb 100–1000 ppb 20–100 ppb	50 ppb 2 ppb	-	surface water	[145]
41	RG-SPCE	Ethinylestradiol		0.5–120 ng L^−1^	0.1 ng L^−1^	humic acid	spiked water sample	[146]
42	Wax-print Ag, pt, Sn sputtered	Cd(II) Zn(II)	SWASV	5–40 μg L^−1^	0.9 μg L^−1^ 1.1 μg L^−1^	Mn(II), Ni(II), Mg(II), Fe(III), Pb((II)	-	[147]
43	CNFs/AuNPs	Mercury	LSV	0.1–1.2 μM	0.03 μM	Cd (II), Pb (II), Cu (II) and Zn (II)	-	[148]
44	Carbon black	AA	CV	-		sodium chloride, sodium bicarbonate, sucrose, citric acid, and sorbitol	dietary supplement	[149]
45	graphite ink	Glucose	-	0.5–50 mM	0.33 mM	-	soft drinks	[150]
46	Silver halide particles	Cysteine Glutathione Homocysteine	Photoreduction	10–100 μM 10–100 μM 20–100 μM	10.0 μM 10.0 μM 7.5 μM	glutamine, glutamic acid, cystine, asparagine, aspartic acid, glycine, histidine, lysine, valine, alanine, and arginine	human blood plasma	[66]
47	3D origami paper-based device	Methyl parathion	Potentiometric	0.1–1.0 nM	0.06 nM	-	-	[151]
48	BR ISE	Bilirubin	Potentiometric	(0.10 μM–1.0 mM	-	-	blood serum	[152]
49	Graphene paper	Kanamycin	Potentiometric	0.05–30 pM	0.05 pM	amoxicillin, ciprofloxacin, tetracycline, and chloramphenicol	milk	[153]
50	DPS containing solid KCl	K^+^ Na^+^ Cl^−^	Potentiometric	-	10^−4.1 ± 0.1^ mol dm^−3^ 10^−3.3 ± 0.1^ mol dm^−3^ 10^−4.1 ± 0.1^ mol dm^−3^	-	sweat	[154]
51	C-dots-AuNPs	Ketamine	Potentiometric	2 × 10^−4^–1 × 10^−3^ mol L^−1^	-	caffeine, glu-curonolactone, histamine, tyramine B-vitamins.	-	[155]
52	Gold/CIM	Cl ^-^	Potentiometric	10^−7^ M–10^−1^ M	-	-	-	[156]
53	G/PEDOT:PSS	Na^+^, K^+^	Potentiometric	10^−4^–1 M 10^−4^–1 M	32 µM for Na^+^ 101 µM for K^+^	-	human urine samples	[157]

BSA—bovine serum albumin; SERS—surface enhanced Raman spectroscopy; POD—paper-based optode devices; Ti3C2Tx/MB—MXene/methylene blue; PBNPs—Prussian blue nanoparticles; CEA—carcinoembryonic antigen; RR—ruthenium red; PGA—poly-galacturonic acid; N-CDs—nitrogen-doped carbon dots; PtNPs—Pt nanoparticles; AgNP/BDD—silver nanoparticle-modified boron-doped diamond; Pd/rGOP—palladium/reduced graphene oxide paper; ZnO NWs—zinc oxide nanowires; CV—cyclicvoltammetry; Zeo–GO—zeolites nanoflakes and graphene-oxide nanocrystals; 5CB—immobilizing 4-cyano-40-pentylbiphenyl; LCs—liquid crystalline molecules; 3D Au NPs/GN—3D Au nanoparticles/graphene; DPV—differential pulse voltammetry; Au@PdPt NPs—trimetallic dendritic Au@PdPt nanoparticles; Cu-MOFs—Cu-based metal–organic frameworks; PAD—paper-based analytical device; AC—acetaminophen; MSQDs—magic-sized quantum dots; FCA—ferrocenecarboxylic acid; 8-OHdG—8-Hydroxy-2′-deoxyguanosine; AFP—Alpha-fetoprotein; FF—Diphenylalanine; EIS—electrochemical impedance spectroscopy; PEDOT: PSS—poly (3,4-ethylenedioxythiophene): poly(styrenesulfonate); CEA—carcinoembryonic antigens; PSA—prostatic specific antigen; ECL—electrochemiluminescence; HbA1c—glycated hemoglobin; MFNCDs—metal oxide hybrid with nitrogen-doped carbon dots; ISOs—carrier-based ion-selective optodes; a-GQDs—Aniline functionalized graphene quantum dots; μTPAD microfluidic thread/paper-based analytical device; HSA—human serum albumin; ME—Magnetoelastic; PEC—photoelectrochemical; ISM—ion-selective membrane; DPS—disposable paper substrate; SWV—square wave voltammetry; CRP—C-reactive protein; cTnI—troponin I; PCT—procalcitonin; SERS—surface enhanced Raman scattering; APTMS-3—aminopropyltriethoxysilane; GA—glutaraldehyde; PPX—poly(chloro-p-xylene); ER—electrochemically reduced graphene; pMFC—paper microbial fuel cell; AA—ascorbic acid.

## Data Availability

Not applicable.
